# Genome-wide transcriptome study in skin biopsies reveals an association of *E2F4* with cadasil and cognitive impairment

**DOI:** 10.1038/s41598-021-86349-1

**Published:** 2021-03-25

**Authors:** Elena Muiño, Olga Maisterra, Joan Jiménez-Balado, Natalia Cullell, Caty Carrera, Nuria P. Torres-Aguila, Jara Cárcel-Márquez, Cristina Gallego-Fabrega, Miquel Lledós, Jonathan González-Sánchez, Ferran Olmos-Alpiste, Eva Espejo, Álvaro March, Ramón Pujol, Ana Rodríguez-Campello, Gemma Romeral, Jurek Krupinski, Joan Martí-Fàbregas, Joan Montaner, Jaume Roquer, Israel Fernández-Cadenas

**Affiliations:** 1 Stroke Pharmacogenomics and Genetics Group, Institut de Recerca de l`Hospital de la Santa Creu i Sant Pau, C/Sant Antoni María Claret 167, Barcelona, Spain; 2 Neurovascular Research Laboratory, Vall d’Hebron Institute of Research, Hospital Vall d’Hebron, Universitat Autònoma de Barcelona, Barcelona, Spain; 3 Stroke Pharmacogenomics and Genetics, Fundació MútuaTerrassa per la Docència i la Recerca, Terrassa, Spain; 4 The Manchester Metropolitan University of All Saints, Manchester, UK; 5 Dermatology Department, Hospital del Mar-Parc de Salut Mar, Barcelona, Spain; 6 Neurology Department, IMIM-Hospital del Mar, Barcelona, Spain; 7 Neurology Department, Hospital Mútua Terrassa, Terrassa, Spain; 8 Neurology Department, Hospital de la Santa Creu i Sant Pau, Biomedical Research Institute Sant Pau (IIB-Sant Pau), Barcelona, Spain; 9 Biomedicine Institute of Seville, IBiS/Hospital Universitario Virgen del Rocío/CSIC, University of Seville, Seville, Spain; 10 Department of Neurology, Hospital Universitario Virgen Macarena, Seville, Spain

**Keywords:** Stroke, Dementia, Headache, Stroke

## Abstract

CADASIL is a small vessel disease caused by mutations in *NOTCH3* that lead to an odd number of cysteines in the EGF-like repeat domain, causing protein misfolding and aggregation. The main symptoms are migraine, psychiatric disturbances, recurrent strokes and dementia, being executive function characteristically impaired. The molecular pathways altered by this receptor aggregation need to be studied further. A genome-wide transcriptome study (four cases paired with three healthy siblings) was carried out, in addition to a qRT-PCR for validation purposes (ten new cases and eight new controls). To study the expression profile by cell type of the significant mRNAs found, we performed an in situ hybridization (ISH) (nine cases and eight controls) and a research in the Single-nuclei Brain RNA-seq expression browser (SNBREB). Pathway analysis enrichment was carried out with Gene Ontology and Reactome. Neuropsychological tests were performed in five of the qRT-PCR cases. The two most significant differentially expressed mRNAs (*BANP,* p-value = 7.23 × 10^–4^ and *PDCD6IP,* p-value = 8.36 × 10^–4^) were selected for the validation study by qRT-PCR. Additionally, we selected two more mRNAs (*CAMK2G,* p-value = 4.52 × 10^–3^ and *E2F4,* p-value = 4.77 × 10^–3^) due to their association with ischemic neuronal death. *E2F4* showed differential expression in the genome-wide transcriptome study and in the qRT-PCR (p = 1.23 × 10^–3^), and it was upregulated in CADASIL cases. Furthermore, higher *E2F4* expression was associated with worse executive function (p = 2.04 × 10^–2^) and attention and information processing speed (IPS) (p = 8.73 × 10^–2^). In situ hibridization showed *E2F4* expression in endothelial and vascular smooth vessel cells. In silico studies indicated that *E2F4* is also expressed in brain endothelial cells. Among the most significant pathways analyzed, there was an enrichment of vascular development, cell adhesion and vesicular machinery terms and autophagy process. E2F4 is more highly expressed in the skin biopsy of CADASIL patients compared to controls, and its expression is present in endothelial cells and VSMCs. Further studies are needed to understand whether E2F4 could be useful as a biomarker, to monitor the disease or be used as a therapeutic target.

## Introduction

CADASIL (cerebral autosomal dominant arteriopathy with subcortical infarcts and leukoencephalopathy, OMIM#125310) is an autosomal dominant inherited small vessel disease (SVD) caused by mutations in the *NOTCH3* gene (19p13). Its real prevalence is unknown, although some studies have shown that up to 4.1/100,000 individuals suffer from CADASIL^1,2^, and as many as 3.2–3.4/1000 general individuals carry cysteine-related missense variant of *NOTCH3*^3,4^.

The extracellular domain (ECD) of Notch3 is constituted by 34 epidermal growth factor-like repeats (EGFr), each of which contains six cysteine residues. Pathogenic mutations lead to an odd number of these cysteines, disrupting disulfide bridge formation and leading to Notch3 protein misfolding, multimerization and aggregation^5–7^. This is the main etiopathogenic hypothesis. However, it is not fully understood. It is thought that this protein aggregation could lead to a toxic gain of function, as seems to occur with TIMP3 and vitronectin (VTN), proteins associated with vessel extracellular matrix^8,9^, or even interfere with autophag ^10^ or cause endoplasmic reticulum stress^11^. Likewise, a proteomic study highlighted the differential protein levels between CADASIL patients and controls regarding extracellular matrix and mitochondrial proteins^12^. However, no robust transcriptomics studies have been carried out in patients with CADASIL.

Because of its systemic nature, the study of different biopsy tissues, such as brain, muscle and skin, has made it possible to examine the histopathological changes that take place in CADASIL for a better comprehension of the disease, such as the loss of endothelial cells, pericytes and vascular smooth muscle cells (VSMC), as well as intercellular or cell–matrix adhesions^13,14^. Actually, the hallmark of the disease, granular osmiophilic materials (GOMs) constituted partly by Notch3 ECD^15^, has a sensitivity of 45–96% and a specificity of 100% for CADASIL diagnosis^16^.

The main symptoms of CADASIL are migraine, psychiatric disorders, recurrent small subcortical infarctions and dementia^17^. Regarding cognitive impairment, 60% of patients aged > 60 years have dementia, and impaired executive function (EF) was detected globally in 87.5% of individuals^18^.

Our aim was to identify mRNA differentially expressed in skin biopsies of CADASIL patients through a genome-wide transcriptome study (GWTS) that could be relevant for understanding the etiopathogenesis of the disease, identifying relevant pathways, therapeutic targets for future studies, as well as to study their association with cognitive performance.

## Methods

This is an observational case–control study to discover differential mRNA expression and enrichment pathways through a genome-wide transcriptome study (GWTS) using microarray technology. For the validation of the significant differentially expressed mRNAs, we performed a qRT-PCR assay with a new cohort of cases and controls, and in situ hybridization (ISH), which will also allow us to locate significant differentially expressed mRNA.

### Subjects

Subjects were selected from “CADAGENIA”, a registry in which patients with mutations in *NOTCH3* have been consecutively recorded since 2017 from different parts of Spain, mostly Catalonia (Hospital Vall d’Hebron and Hospital del Mar, Barcelona). For matching purposes, control relatives (such as spouses or siblings) without a known *NOTCH3* mutation were asked to enroll in the registry to avoid any potential bias due to differences between cases and controls, as well as other healthy volunteers.

Epidemiological data, blood analyses, cognition and neuroimaging profiles and skin biopsies were registered.

The inclusion criteria for cases for this differential expression study were: (1) age > 17 years, (2) having a cysteine-affecting *NOTCH3* missense mutation (CNMM), and (3) having a skin biopsy available. The exclusion criteria were: (1) age < 18 years, (2) having a *NOTCH3* mutation other than CNMM, and (3) not having a skin biopsy available.

The inclusion criteria for controls were: (1) age > 17 years, and (2) agreeing to have a skin biopsy. The exclusion criteria were: (1) age < 18 years, (2) having a known *NOTCH3* mutation, and (3) not having a skin biopsy available.

As additional inclusion criteria for the GWTS, CADASIL patients and controls had to be matched with family members. For the qRT-PCR and ISH assays, this criterion was not needed. None of the controls was related to the cases in the qRT-PCR or in the ISH study.

For ISH, all nine cases had been studied with the qRT-PCR. Of the eight controls, seven had been studied with qRT-PCR and one with the GWTS.

### Variables

Detailed clinical-epidemiological data were collected from each patient, including age; sex; vascular risk factors, such as hypertension defined as two measures on different days with blood pressure exceeding 140/90 mmHg or taking antihypertensive treatment; diabetes mellitus (DM), defined as basal glycemia in venous plasma ≥ 126 mg/dl, 2-h post-load plasma glycemia ≥ 200 mg/dl or HbA1c ≥ 6.5% or taking antidiabetic treatment; dyslipidemia; smoking habits; and type of mutation.

The cognitive profile was determined in patients with *NOTCH3* mutations by means of a complete neuropsychological examination. The evaluated cognitive domains included: verbal memory, working memory, executive function, attention and information processing speed, motor speed and dexterity, and visuoconstructional skills.

For global cognition, the Montreal Cognitive Assessment (MOCA) was used as a screening test. Verbal memory was evaluated through the short-term total learning and delayed recall subtests from the Wechsler memory scale-III (WMS-III) word list. Working memory was determined by the forward and backward digits subtests from the Wechsler Adult Intelligence Scale (WAIS-III). EF was assessed by means of: phonetic (letters “P”, “M” and “R”) and semantic category (animals) verbal fluencies, the Stroop Color-Word test—number of words—and the Trail Making Test part B (TMT-B)—execution time. Attention and information processing speed (IPS) were evaluated through the Symbol Digit Modalities Test (SDMT), Stroop Word and Color tests—number of words—and the Trail Making Test part A (TMT-A)—execution time. Motor speed and dexterity were rated by the Purdue Pegboard test, considering the dominant, non-dominant and both-hand trials. Visuoconstructional skills were evaluated by means of the block designs subtest from the WAIS-III.

Raw scores were adjusted into Z-scores by age and years of education following Spanish normative data^19–21^. A higher adjusted Z-score indicates a better performance in all cases. We calculated cognitive domain indices by averaging the adjusted scores within each domain.

### RNA extraction

A 6-mm skin punch biopsy was obtained for each participant in the study. The homogenization of the tissue was carried out with the *TissueRuptor* (Quiagen) and the RNA was extracted with a RNeasy Plus Micro Kit (Quiagen), following the manufacturer’s instructions.

### Genome-wide transcriptome study

From each sample, 10 ng of total RNA was used as the starting material. The quality of the isolated RNA was measured previously by capillary electrophoresis using a NanoChip (Bioanalyzer 2100, Agilent). Single-stranded cDNA suitable for labeling was generated from the total RNA using the GeneChip WT Pico Reagent Kit (Thermo Fisher Scientific) according to the manufacturer’s instructions. This kit makes it possible to generate robust expression profiles from as little as 100 pg of total RNA (10 cells). Purified sense-strand cDNA was fragmented, labeled and hybridized to the arrays using the GeneChip Hybridization, Wash and Stain Kit from the same manufacturer. Finally, Affymetrix Human Clariom S Pico Assay was the microarray used to study the expression. After array scanning, raw data quality control was performed to check the overall performance of the processing.

### qRT-PCR

For this assay, we selected the two most significantly differentially expressed mRNAs from the GWTS (p-value < 10^–3^). As CADASIL is an arteriopathy that leads to brain hypoxemia, we wanted to select the genes from the GWTS that were related to neuronal ischemia in order to show a possible link and that belonged to the top fifteen most significant differentially expressed mRNAs. Therefore, we conducted a bibliographic search in PubMed with the term “(ischemi*[Title/Abstract]) AND *gene*[Title/Abstract]”.

As previously reported^22^, mRNA levels were measured by qRT-PCR using TaqMan fluorogenic probes (see Supplementary Table I for those used in this study) on a 7500 Real-Time PCR System (Applied Biosystems, CA, USA). PPIA expression was used to normalize the results, as has been described previously^22^.

qRT-PCR was performed using a standard TaqMan PCR kit protocol consisting of 20 µl of PCR mix, including 5 µl of cDNA, 10 µl of 2 × TaqMan Universal PCR Master Mix (P/N: 4304437, Applied Biosystems, Foster city, CA, USA), 1 µl of TaqMan gene expression assay and 4 µl of water. Reactions were performed in two 384-well plates at 50 °C for two min and at 95 °C for 10 min, followed by 40 cycles at 95 °C for 15 s and 60 °C for one min. All reactions were run in triplicate and analyzed using the RQ App on Thermo Fisher Connect, following standard quality controls to assess the samples.

The results were a relative quantification (RQ) between the cycles of each sample relative to a single calibrator control sample.

### ISH

Formalin-fixed human skin tissues were embedded in paraffin, cut at 3–4 μm and stained with RNAscope Probe—Hs-E2F4—E2F Transcription Factor 4 probe (898351, Bio-techne) using RNAscope Intro Pack 2.5 HD Reagent Kit Red (322350, Bio-techne).

Thereafter, anti-CD31 antibody (ab28364, Abcam), used as a marker of vessels23 and the mouse monoclonal alpha smooth muscle Actin antibody [1A4] (αSMA) (ab7817, Abcam) used as a marker of myofibroblasts/fibroblasts^23^ and VSMC, were used for immunofluorescence. DAPI staining was used to stain all nuclei.

Full images of sections were acquired with a NanoZoomer-2.0 HT C9600 scanner (Hamamatsu) at 20× magnification. QuPAth open software was used to perform image analysis^24^. Region of interests (ROIs) were selected manually in all the tissue sections, including the full dermis, and excluding the epidermis and sebaceous/sudoriferous glands.

A positive pixel count algorithm was used to detect CD31 cells or αSMA, and they were segmented in positive or negative. Subcellular detection was used afterwards to detect the number of *E2F4* spots, which were segmented in dots (up to 6 µm^2^) or clusters (> 6 µm^2^).

For the quantitative analysis we only considered those CD31+ cells that are surrounded by αSMA+ cells and vice versa, and can therefore be said to be EC and VSMC.

### Statistical methods

Statistical and bioinformatics analyses were performed using custom scripts in R language, version 3.6.0 (R Core Team, 2019), with common Bioconductor packages. For the GWTS, after following a standard quality control, the Robust Multi-array Average (RMA) algorithm was used for pre-processing transcriptome data in order to perform background adjustment, normalization and summarization of the probe set expression values. Then, genes whose standard deviation (SD) was below the 65 percentiles of all the SD values, without a known Entrez Gene database identifier and without a valid annotation to the Gene Ontology database, were filtered out from the whole dataset and finally 6485 genes were considered for the statistical analysis. Selection of differentially expressed elements was based on a linear model analysis with empirical Bayes modification for the variance estimates. To deal with the false‐discovery rate derived from multiple test comparisons, p-values were adjusted with the Benjamini and Hochberg method^25^, considering genes with an adjusted p-value < 0.05 to be statistically significant.

The two most significant differentially expressed mRNAs from the GWTS (p-value < 10^–3^) were evaluated in the replication cohort by qRT-PCR. Another two significant differentially expressed mRNAs from the top fifteen that were associated with ischemic neuronal death were also analyzed.

As the inclusion of outlying values could lead to erroneous interpretations^26^, a box plot was performed for their identification. We used the “ggbetweenstats” function from the “ggstatsplot” package library. To know whether the outliers were statistically significant, and therefore that sample should be excluded, a Dixon's Q test was performed with the “dixon.test” function from the “outliers” package.

A p-value < 0.05 was considered statistically significant, after Bonferroni multivariable test correction, in the validation analysis.

For the ISH, a box plot was created with the “ggplot2” package library. Samples with values below quartile 1 minus 1.5 times the interquartile range, or above quartile 3 plus 1.5 times the interquartile range, were considered outliers for that analysis and therefore removed. A p-value < 0.05 was considered statistically significant.

To assess statistical significance, Fisher's Exact Test was used for categorical variables and a Mann–Whitney U test was used for not normally distributed continuous variables or ordinal variables. The T-test was used for normal and homoscedastic continuous variables. Pearson’s test was used to study the correlation between normal numeric variables.

### Expression profile

Brain expression of the mRNAs replicated in the qRT-PCR was studied using the GTEx Portal (https://gtexportal.org/home/) and expression by brain cell type was studied in the Single-nuclei Brain RNA-seq expression browser (http://ngi.pub/snuclRNA-seq/).

### Enrichment pathways analysis

The analysis of biological significance was based on gene set enrichment analysis (GSEA), which makes it possible to detect situations where all genes in a predefined set change in a small but coordinated way^27^. The analysis has been performed over two annotation databases: the “Gene Ontology”(GO) and the Reactome Pathway Knowledge base^28^.

All the filtered genes analyzed in the GWTS were ranked by log2 fold change and were used in the analysis. For GO, analysis was performed regarding biological process (BP), cellular component (CC) and molecular function (MF). GO terms and Reactome Pathways were considered enriched with a raw p-value under 0.01.

An enrichment map of the top 60 terms or pathways found for each comparison was performed (for GO terms enriched in the category of Molecular Function there were just 11 terms, and 37 for the enriched pathways). This map groups gene ontology (GO) terms/pathways by similarity. Nodes are colored by p-value and their size reflects the number of genes found in that term.

### Ethical issues

All protocols were carried out in accordance with the guidelines and national regulations, being approved by the local ethics committee (Hospital del Mar and Hospital Vall d’Hebron). A written informed consent document was provided before any study procedure was performed and it was signed by the patient or representative.

## Results

### Differential expression study

#### GWTS study

For the GWTS study, we had four CADASIL patients corresponding to three different families, and three sibling controls without CNMM, one per family, in the CADAGENIA registry. These seven patients constituted the discovery cohort. Ten CADASIL patients and eight controls matched by age and sex constituted the validation cohort; blood relatives were not mandatory. No statistically significant differences between cases and controls were observed in age, sex, smoking habits, hypertension, diabetes mellitus, dyslipidemia, migraine, psychiatric disease, stroke or dementia (Table 1).

**Table 1:** Clinical-epidemiological features of the GWTS, qRT-PCR and ISH assay cohorts.

	GWTS cases (n = 4)	GWTS controls (n = 3)	p-value	qRT-PCR cases (n = 10)	qRT-PCR controls (n = 8)	p-value	ISH cases (n = 9)	ISH controls (n = 8)	p-value
Age (mean ± SD, years)	52 ± 15	58 ± 16	0.21	49 ± 15	42 ± 10	0.31	45 ± 11	44 ± 7	0.63
Female	75% (3/4)	67% (2/3)	1	50% (5/10)	50% (4/8)	1	56% (5/9)	38% (3/8)	0.64
Smoker	25% (1/4)	33% (1/3)	1	10% (1/10)	25% (2/8)	0.56	11% (1/9)	50% (4/8)	0.13
HTN	0%	33% (1/3)	0.43	36% (3/10)	13% (1/8)	0.59	22% (2/9)	13% (1/8)	1
DM	0%	0%	1	10% (1/10)	0%	1	0%	0%	1
Dyslipidemia	25% (1/4)	0%	1	30% (3/10)	25% (2/8)	1	22% (2/9)	25% (2/8)	1
Migraine	50% (2/4)	67% (2/3)	1	30% (3/10)	25% (2/8)	0.19	56% (5/9)	29% (2/7)	0.36
Psychiatric disease	25% (1/4)	33% (1/3)	1	60% (6/10)	25% (2/8)	0.61	66% (6/9)	38% (3/8)	0.35
Stroke	0%	0%	1	40% (4/10)	0%	0.25	33% (3/9)	0%	0.21
Dementia	0%	0%	1	0%	0%	1	0%	0%	1

*HTN* hypertension, *DM* diabetes mellitus.

**Figure 1:**
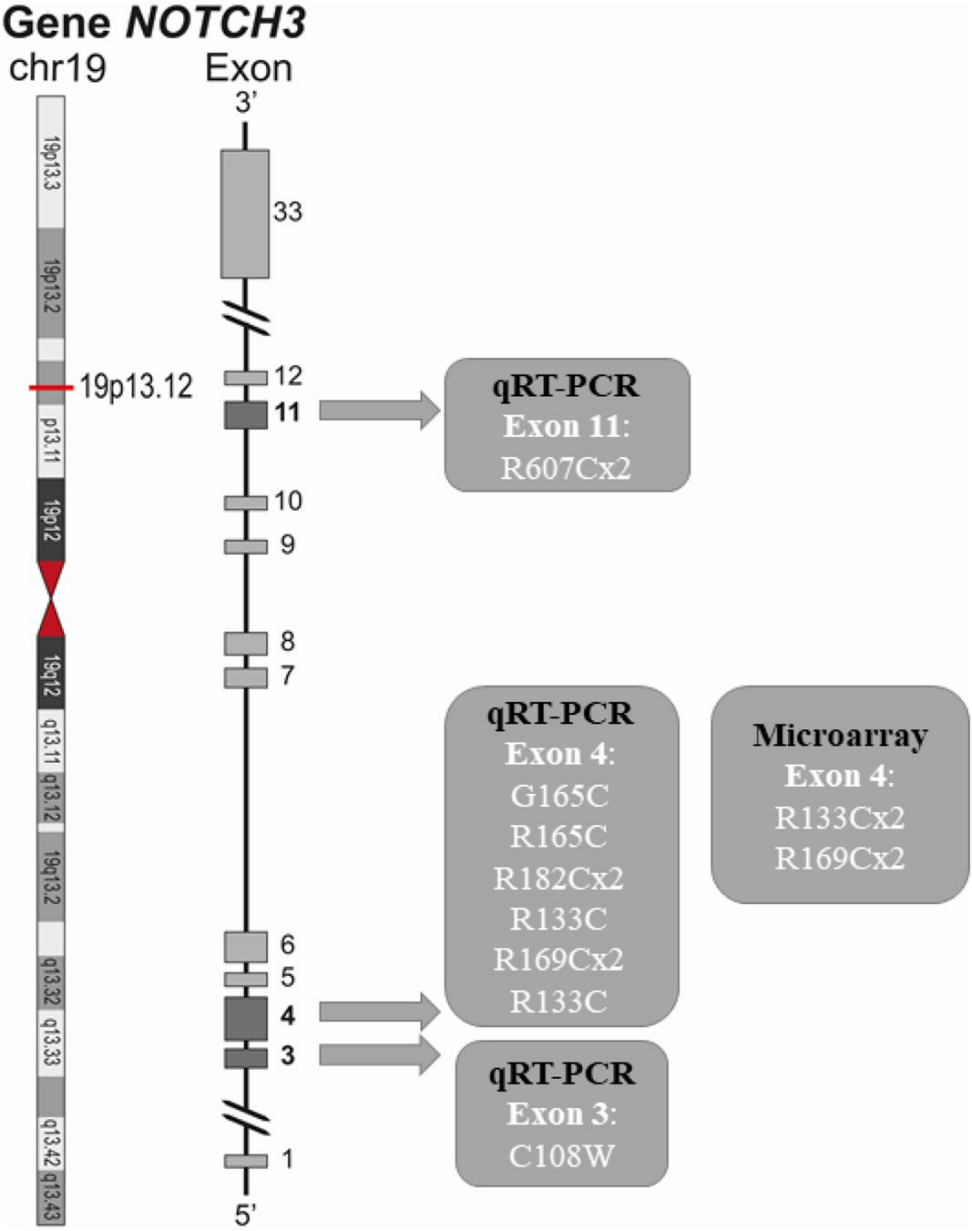
Distribution of mutations from the cases of the GWTS and qRT-PCR studies. *Chr* chromosome.

For the distribution of the mutations in the cases of the Discovery/Validation analyses, see Fig. 1.

The GWTS did not show statistically significant differential expression after Benjamini and Hochberg correction (Table 2).

**Table 2:** Top fifteen most differentially expressed mRNAs from the Genome-Wide Transcriptome study.

Gene symbol	Gene name	logFC	Raw p-value
BANP	BTG3 associated nuclear protein	− 1.40	7.23 × 10^–4^
PDCD6IP	Programmed cell death 6 interacting protein	− 1.20	8.36 × 10^–4^
TEM176A	Transmembrane protein 176A	− 1.23	1.87 × 10^–3^
C16orf70	Chromosome 16 open reading frame 70	− 1.10	2.37 × 10^–3^
JCHAIN	Joining chain of multimeric IgA and IgM	− 1.46	2.87 × 10^–3^
CCDC149	Coiled-coil domain containing 149	− 1.00	3.57 × 10^–3^
GLT8D2	Glycosyltransferase 8 domain containing 2	− 1.76	3.95 × 10^–3^
PPIE	Peptidylprolyl isomerase E	0.94	3.97 × 10^–3^
ZFP64	ZFP64 zinc finger protein	− 0.99	4.40 × 10^–3^
CAMK2G	Calcium/calmodulin dependent protein kinase II gamma	− 0.85	4.52 × 10^–3^
CPA3	Carboxypeptidase A3	− 1.10	4.66 × 10^–3^
E2F4	E2F transcription factor 4	0.89	4.77 × 10^–3^
ZBTB24	Zinc finger and BTB domain containing 24	− 0.91	4.95 × 10^–3^
AP3M2	Adaptor related protein complex 3 subunit mu 2	− 1.26	4.96 × 10^–3^
SFMBT2	Scm-like with four mbt domains 2	− 0.98	5.37 × 10^–3^

*logFC* log fold change.

#### qRT-PCR study

The two genes with the smallest p-values (p-value < 10^–3^) (Table 1): *BANP,* p-value = 7.23 × 10^–4^ and *PDCD6IP,* p-value = 8.36 × 10^–4^, both downregulated in cases; and the two most relevant mRNAs associated with ischemic neuronal death from the top fifteen most significant differentially expressed mRNAs: *CAMK2G,* downregulated in cases (p-value = 4.52 × 10^–3^), and *E2F4,* upregulated in cases (p-value = 4.77 × 10^–3^), were selected for a second study using qRT-PCR in a new cohort of ten CADASIL patients and eight controls.

**Figure 2:**
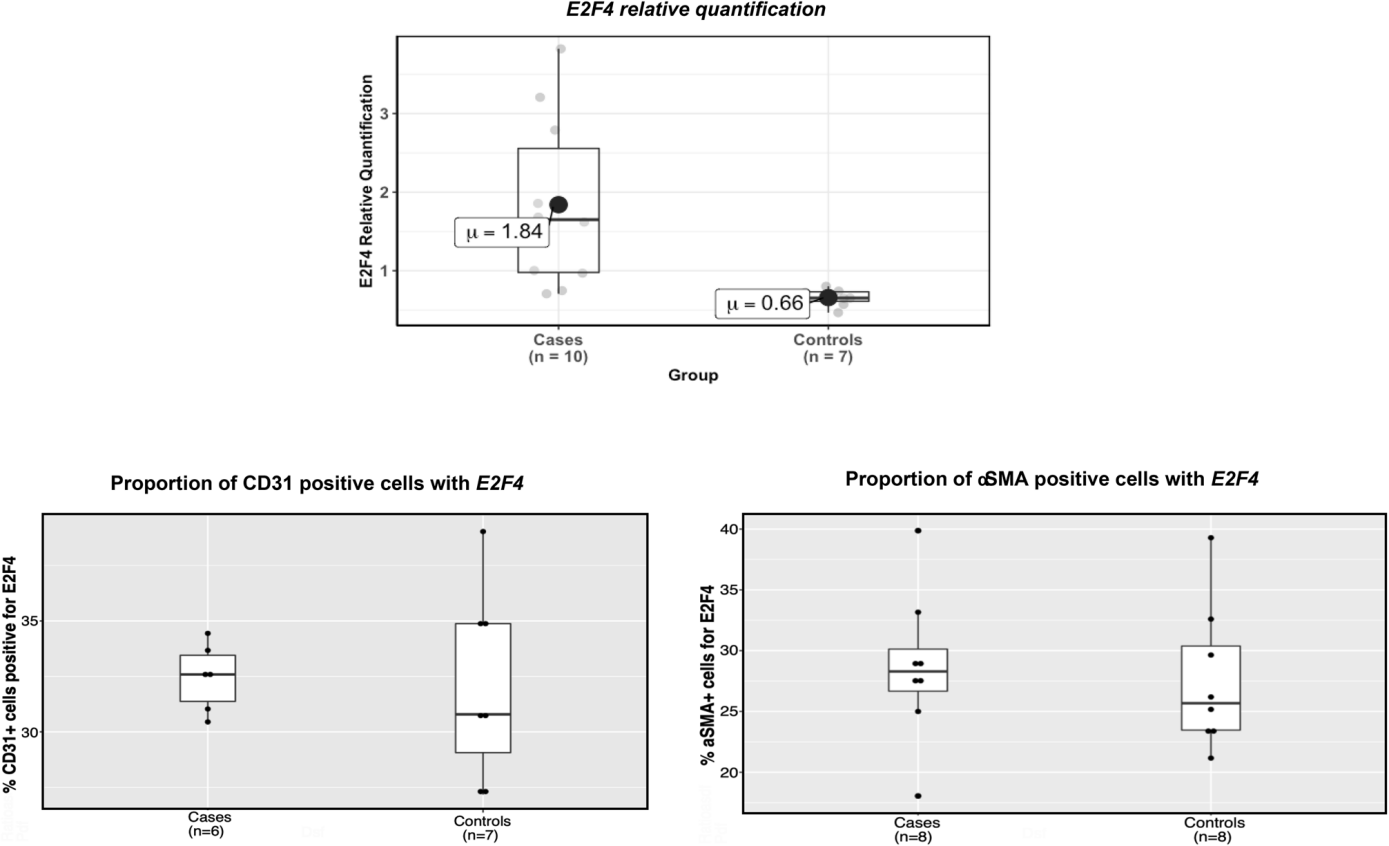
*E2F4* expression. (**A**) *E2F4* relative quantification box plot for cases and controls from the qRT-PCR experiment. (**B**) Proportion of CD31 positive cells with *E2F4* expression. (**C**) Proportion of αSMA positive cells with *E2F4* expression.

In the qRT-PCR assay (Table 3), *E2F4* mRNA levels were significantly higher in CADASIL patients compared with controls after Bonferroni correction (relative quantification of 1.84 ± 1.09 in cases and 0.66 ± 0.11 in controls, raw p-value = 1.23 × 10^–3^; Bonferroni threshold: 1.25 × 10^–2^; see Fig. 2A).

**Table 3:** Mean, standard deviation and p-values of the mRNAs from the qRT-PCR assay.

	Cases RQ (mean ± SD)	Controls RQ (mean ± SD)	Raw p-value
BANP (10 cases, 7 controls)	1.09 ± 0.36	1.05 ± 0.39	1
PDCD6IP (9 cases, 8 controls)	1.72 ± 0.81	1.64 ± 0.61	0.884
CAMK2G (10 cases, 8 controls)	1.03 ± 0.48	0.87 ± 0.17	0.12
E2F4 (10 cases, 7 controls)	1.84 ± 1.09	0.66 ± 0.11	1.23 × 10^–3^

Ten cases and eight controls were evaluated. The table shows the final size after qRT-PCR quality controls and removal of significant outliers.

*RQ* relative quantification, *SD* standard deviation.

#### ISH

This cohort was constituted by nine cases and eight controls. Sixteen of them were chosen from the GWTS and one from the qRT-PCR study, due to their availability for the ISH. None of the controls was related to the cases.

**Figure 3:**
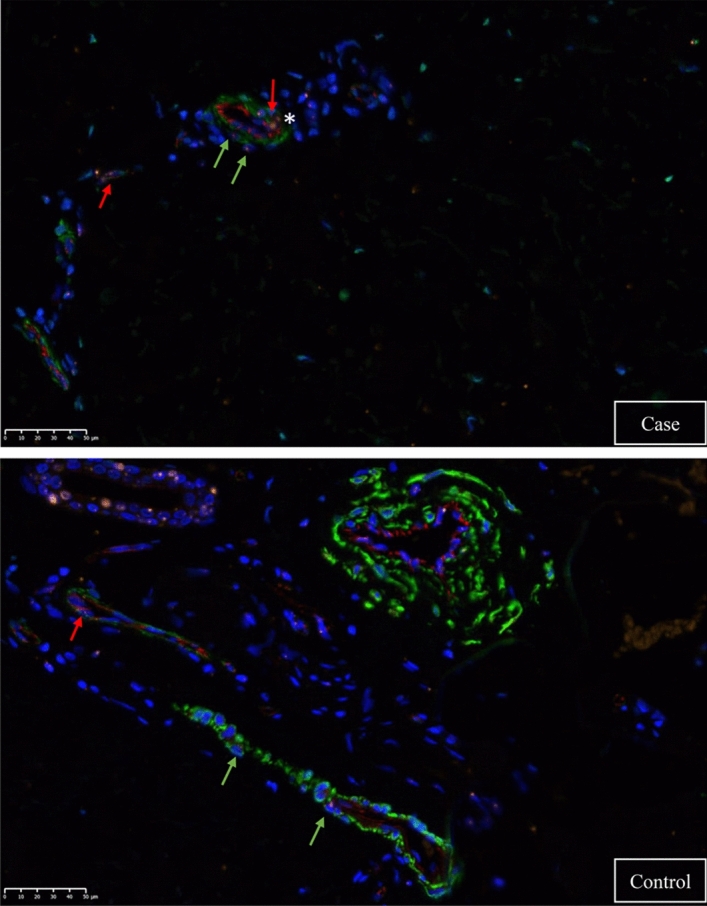
In situ hybridization of *E2F4* in dermis. Fluorescence for *E2F4* is shown in orange, for CD31 in red and for αSMA in green. The image above corresponds to a case and the image below to a control. The red arrows indicate *E2F4* positivity in a CD31-positive cell. Green arrows indicate *E2F4* positivity in an SMA-positive cell. The asterisk indicates an *E2F4* cluster.

ISH with E2F4 probe revealed positive labeling in the epidermis, some cells of the dermis and some cells of the skin adnexa. *E2F4* dots were located on nuclei and in the cytosolic subcellular compartment (Fig. 3).

CD31 positive labeling presented a membranous pattern and was seen in some cells of the dermis. αSMA positive cells presented cytoplasmic labeling and were seen in cells surrounding the CD31 positive cells and below the epithelial cells of sebaceous/sudoriferous glands.

We did not find significant difference of *E2F4* expression level between cases and controls (Table 4 and Fig. 2).

**Table 4:** Statistical summary of the in situ hybridization.

		Cases median (Q1–Q3)	Controls median (Q1–Q3)	p-value
EC	% Positive E2F4 (6 cases, 7 controls)	33% (31–33)	31% (29–35)	0.95
	% Cells with dots (7 cases, 6 controls)	32% (31–33)	31% (29–33)	0.53
	% Celss with clusters (6 cases, 8 controls)	7% (6–8)	6% (5–9)	0.66
VSMC	% Positive E2F4 (8 cases, 8 controls)	28% (27–30)	26% (24–30)	0.57
	% Cells with dots (8 cases, 8 controls)	29% (27–31)	26% (21–29)	0.44
	% Celss with clusters (7 cases, 8 controls)	30% (26–31)	26% (21–29)	0.12

For endothelial cells and VSMC positive cells, there are: (1) Proportion of positive cells with *E2F4*, (2) Proportion of cells with *E2F4* dots, and (3) Proportion of cells with *E2F4* clusters.

Samples with values below quartile 1 minus 1.5 times the interquartile range, or above quartile 3 plus 1.5 times the interquartile range, were considered outliers for that analysis and therefore removed.

*EC* endothelial cells, *VSMC* vascular smooth muscle cells.

#### Cognition evaluation

To study the relationship between *E2F4* and neuropsychological performance, five CADASIL patients with qRT-PCR data were analyzed. Each neuropsychological domain (ND) was altered in > 50% of the patients, except visuoconstructional function. See Supplementary Table II, Supplementary Fig. I.

A significant association was observed with EF, correlation = − 0.93, p-value = 2.04 × 10^–2^, and a tendency was observed in attention and IPS, correlation = − 0.82, p = 8.73 × 10^–2^, adjusted by age and educational level (Table 5). Higher *E2F4* expression was associated with a worse score in the EF and IPS tests. The other cognitive domains were not associated with *E2F4*.

**Table 5:** p-value of the association between E2F4 levels and domain alteration, adjusted by age and educational level.

Neuropsychological domain	Cor	p-value
Executive function	− 0.93	2.04 × 10^–2^
Attention and information processing speed	− 0.82	8.73 × 10^–2^
Motor speed	− 0.21	7.40 × 10^–1^
Visuoconstructional skills	− 0.49	4.05 × 10^–1^
Verbal memory	− 0.16	7.93 × 10^–1^
Working memory	0.16	7.97 × 10^–1^

*Cor* correlation.

### Expression profile

The GTEx Portal showed *E2F4* is expressed in the brain, as well as in other tissues (Supplementary Fig. II), and the SNBREB showed that it is also expressed in brain endothelial cells (Supplementary Fig. III).

### Pathway enrichment analysis

GO terms and Reactome Pathways were considered enriched with a raw p-value under 0.01, since none of the analyses reached a significant p-value after correction for multiple comparisons. See Supplementary Figs. IV–V and Supplementary Tables III–IV.

In the BP analysis (Supplementary Fig. IV), we observed the network related to vascular development, and catabolic and autophagy processes, and in the CC analysis (Supplementary Fig. V), the enrichment in vesicular machinery and cell adhesion terms.

## Discussion

*E2F4* mRNA was associated with CADASIL patients and was upregulated in the skin biopsies of cases vs controls in the GWTS and the qRT-PCR study. The expression profile showed that *E2F4* is widely expressed (GTEx portal), including endothelial cells in the brain (SNBREB database). Moreover, our ISH confirmed *E2F4* expression in endothelium and VSMCs, cells that are characteristically affected in the disease.

We did not find significant difference of *E2F4* expression level between cases and controls in our ISH. The lack of statistical significance could be due to sampling bias, limited number of slices and limited number of vessels in a slice, among others.

Besides, *E2F4* expression was additionally inversely correlated with EF and attention and IPS, which are the principal cognitive functions that are altered in CADASIL^29^.

Whether E2F4 might be useful as a biomarker of early detection of cognitive impairment and monitoring of the course of the disease should be addressed by increasing the number of patients. For practical use, it is necessary to identify blood biomarkers reflecting E2F4 expression level in skin biopsy.

The E2F4 protein belongs to the E2F family of transcription factors and plays a crucial role in controlling the cell cycle. *E2F4* has expression in endothelial cells, as we have shown in the ISH, and it is necessary for its correct migration^30^. A diminished expression of *E2F4* attenuates the endothelial cell migration, and its subsequent overexpression could rescue normal endothelial migration^30^.

*E2F4* is also expressed in VSMC^31^, also evidenced in our ISH. Actually, it is involved in the process of intimal hyperplasia (IH), which is the proliferation of VSMCs in the media and their migration into the tunica intima of the vessel. Mice lacking E2F4 exhibit increased IH following arterial damage^31^.

Both, endothelial cells and VSMC, are key cells in the aetiopathology of this disease, which highlights the relevance of the finding of *E2F4* as a mRNA differentially expressed. These cells have been found to be altered in CADASIL histopathological studies^13,32,33^ and they are involved in blood flow regulation, a mechanism that has been seen to be altered in numerous studies focusing on this disease^34–36^.

Besides, E2F4 have been related to neuronal survival in ischemic situations. Previous studies observed that primary cerebellar granule neurons (CGNs) overexpressing *E2F4* vs controls had higher survival after an ischemic insult^37^. Nevertheless, E2F4 levels decreased after hypoxia in non-infected CGNs. Overexpression of E2F4 had no effect on neuronal viability in the absence of ischemia.

E2F4 is also part of a complex containing Smad3, which acts as a transducer of transforming growth factor-β (TGFβ) signals^38^. TGFβ is a protein related to hereditary SVDs^39^ such as CARASIL, caused by *HTRA1* gene mutations. HTRA1 is also associated with LTBP-1 and they regulate bioavailability of TGFβ^40^. Importantly, these two proteins have been associated with Notch3 ECD deposits^41^ and HTRA1 has shown less activity in CADASIL patients^42^.

Whether the elevated expression of *E2F4* is due to a compensatory mechanism for a lack of protein production or whether the protein is actually elevated, is still unclear.

In view of the above, it would seem more likely that there was an increase in E2F4 protein levels. In CADASIL, there is a loss of VSMC^13,14^, and the absence of E2F4 activity leads to increased proliferation of VSMC^31^. In addition, increased mRNA and protein levels of E2F4 could indicate an overactivation of the TGFβ pathway in CADASIL patients, a molecule widely associated with SVD^39^ and fibrosis^43^. Actually, TGFβ1 is implicated in cell proliferation, differentiation, apoptosis, autophagy and extracellular matrix protein production^44^.

This is a study of gene expression through microarray technology and validation by real-time quantitative reverse transcription polymerase chain reaction (qRT-PCR)^22^.

So far, proteomic studies in CADASIL have identified an enrichment of extracellular matrix proteins, mitochondrial proteins^12^, proteins associated with degradation and folding, contraction of VSMC, and cellular stress^45^. They have provided valuable information for understanding the disease, such as finding the enrichment of HTRA1 and colocalizing it with Notch3 ECD deposits, linking the molecular pathways between CADASIL and CARASIL^12^. They have also made it possible to see that TIMP3 and vitronectin (VTN) are sequestered by Notch3 ECD deposits^46^, leading to studies that showed that high levels of TIMP3 and VTN play a role in CADASIL, producing diverging influences on CBF deficits and white matter lesions^8^.

Transcriptomic studies are a complementary approach that analyzes the different potential mechanisms associated with CADASIL. Moreover, transcriptomics could be highly correlated with clinical traits compared with protein levels in mice^47^.

Despite the lack of significant results after correction by multiple tests, the pathway studies have shown an increase in the biological processes related to vascular development, which is remarkable because CADASIL is a systemic arteriopathy caused by *NOTCH3*, a gene related to vascular morphogenesis.

GO analysis have also shown an increase in cellular component terms related to cell adhesion. Additionally, it should be noted that histopathological studies of CADASIL have shown a change in or loss of cellular junctions between VSMC or the adjacent extracellular matrix and endothelial adhesion^13,14,33^.

Moreover, the pathway analysis has shown an enrichment of component cells terms related to vesicular machinery and the biological process of autophagy. Several articles have pointed out that this cellular clearance pathway could be impaired in CADASIL, causing a deficiency in the elimination of Notch3 aggregates^10,48,49^.

### Limitations

This study has several limitations. Firstly, the small sample size due to the low frequency of the disease in question. Matching the patients by age and sex and by family in the GWTS allowed us to minimize biases in the interpretation of the results.

Secondly, the target organ in CADASIL is the brain, but samples were obtained from skin tissue. Nevertheless, histopathological studies of skin biopsies have shown the typical hallmarks of the disease and have been used to understand its etiopathogenesis^13,32,33^. Besides, post-mortem brain tissue can be problematic for transcriptomic analysis due to apoptotic and necrotic processes that change the gene expression, causing bias in omics experiments.

Thirdly, the GWTS study did not present significant differentially expressed mRNAs associated with CADASIL after adjusting for multiple comparisons. Massive data studies in low prevalence diseases such as CADASIL, which include different types of cells, may have the inconvenience of presenting less power to detect statistical significance when small differences exist, even if they are determinant. Choosing the mRNAs most significantly associated with CADASIL in the GWTS for evaluation by the qRT-PCR technique may be a useful and valid approach. Genetic studies in CADASIL have also had to resort to the use of non-significant data to deal with this lack of power, subsequently obtaining data of scientific interest. For example, the creation of a polygenic score from the most significant SNPs associated with WMH volume in CADASIL patients, suggesting that multiple SNPs with small effects modify the total WMH load in patients with CADASIL, rather than SNPs with larger effects^50^.

Finally, as in the skin there are lymphatic blood vessels with endothelial cells, we cannot totally exclude that some of the CD31 positive cells found in the ISH, might be also labelling those. In the same way, there are other cells in the skin that might be positive to αSMA, such as fibroblast. Nevertheless, lymphoid vessels do not present the smooth muscle layer around them, therefore it can be assumed that the vast majority of CD31 immunostained cells corresponded to endothelial cells and the αSMA positive cells surrounding these endothelial cells were VSMC. We have checked histologically when doing the image analysis quantification that CD31 positive cells were surrounded by αSMA positive cells.

## Conclusions

Our results showed higher levels of *E2F4* mRNA in CADASIL skin biopsies and the highest levels of expression were associated with the worst EF and attention and IPS in five of the qRT-PCR CADASIL cases.

E2F4 is a protein expressed in endothelial and VSMC (confirmed in our ISH), controlling the migration of the former and the proliferation and migration of the latter, which are cells that are characteristically affected in the disease. Besides, it is a protein that is related to neuronal survival in ischemic conditions and the TGFβ pathway. *E2F4* should be studied further to clarify whether its expression levels might help to monitor the disease and cognitive status, making it important for future clinical trials or even for therapeutic targeting. Further studies are needed to elucidate the role of *E2F4* in CADASIL.

## Supplementary Information

Supplementary Information.

## Data Availability

The datasets used and analyzed in the present study are available from the corresponding author on reasonable request.

## Abbreviations

CADASIL: Cerebral autosomal dominant arteriopathy with subcortical infarcts and leukoencephalopathy
SVD: Small vessel disease
ECD: Extracellular domain
EGFr: Epidermal growth factor-like repeats
VTN: Vitronectin
qRT-PCR: Real-time quantitative reverse transcription polymerase chain reaction
GOMs: Granular osmiophilic materials
EF: Executive function
GWTS: Genome-wide transcriptome study
ISH: In situ hybridization
CNMM: Cysteine-affecting *NOTCH3* missense mutation
MOCA: Montreal Cognitive Assessment
WMS-III: Wechsler Memory Scale-III
WAIS-III: Wechsler Adult Intelligence Scale
TMT-B: Trail Making Test part B
TMT-A: Trail Making Test part A
RQ: Relative quantification
RMA: Robust multi-array average algorithm
SD: Standard deviation
IPS: Information processing speed
αSMA: Alpha smooth muscle actin
ROI: Region of interest
GSEA: Gene set enrichment analysis
GO: Gene Ontology
BP: Biological process
CC: Cellular component
MF: Molecular function
TGFβ: Transforming growth factor-β
IH: Intimal hyperplasia
