# Supplementary material for: Genome-wide transcriptome study in skin biopsies reveals an association of E2F4 with cadasil and cognitive impairment
Source: Sci Rep. 2021 Mar 25;11:6846. doi: 10.1038/s41598-021-86349-1 (PMC7994794; doi:10.1038/s41598-021-86349-1)
Supplement: Supplementary file 1 — Supplementary Information. [file 41598_2021_86349_MOESM1_ESM.docx]

**SUPPLEMENTAL MATERIAL**

GENOME-WIDE TRANSCRIPTOME STUDY IN SKIN BIOPSIES REVEALS AN ASSOCIATION OF *E2F4* WITH CADASIL AND COGNITIVE IMPAIRMENT

Elena Muiño PhD^1^, Olga Maisterra PhD^2^, Joan Jiménez-Balado PhD^2^, Natalia Cullell MSc^1,3^, Caty Carrera MD^1^, Nuria P Torres-Aguila MSc^1^, Jara Cárcel-Márquez MSc^1^, Cristina Gallego-Fabrega^1,3^, Miquel LLedós MSc^1^, Jonathan González-Sánchez MSc^3,4^, Ferran Olmos-Alpiste MD^5^, Eva Espejo^5^, Álvaro March MD^5^, Ramón Pujol PhD^5^, Ana Rodríguez-Campello PhD^6^, Gemma Romeral^6^, Jurek Krupinski PhD^7^, Joan Martí-Fàbregas PhD^8^, Joan Montaner PhD^4,9^, Jaume Roquer PhD^6^, Israel Fernández-Cadenas PhD^1*^.

1. Stroke Pharmacogenomics and Genetics group. Institut de Recerca de l`Hospital de la Santa Creu i Sant Pau. Spain.

2. Neurovascular Research Laboratory. Vall d’Hebron Institute of Research, Hospital Vall d’Hebron. Universitat Autònoma de Barcelona. Spain.

3. Stroke Pharmacogenomics and Genetics. Fundació MútuaTerrassa per la Docència i la Recerca. Spain.

4. The Manchester Metropolitan University of All Saints. UK

5. Dermatology Department. Hospital del Mar-Parc de Salut Mar. Spain.

6. Neurology Department. IMIM-Hospital del Mar. Spain.

7. Neurology Department. Hospital Mútua Terrassa. Spain.

8. Neurology Department. Hospital de la Santa Creu i Sant Pau. Spain.

9. Biomedicine Institute of Seville, IBiS/Hospital Universitario Virgen del Rocío/CSIC/University of Seville & Department of Neurology, Hospital Universitario Virgen Macarena. Spain.

Corresponding author:

Israel Fernández Cadenas: Stroke Pharmacogenomics and Genetics group. Institut de Recerca de l`Hospital de la Santa Creu i Sant Pau. C/Sant Antoni María Claret 167. Spain.

Email: [israelcadenas@yahoo.es](mailto:israelcadenas@yahoo.es)

Telephone number: +34 937 365 050

Number of figures and tables: one figure, two tables.

Table of contents

[1. Supplemental Tables 5](#_Toc66867166)

[Supplemental Table I. 5](#_Toc66867167)

[Supplemental Table II. 6](#_Toc66867168)

[Supplemental Table III. 7](#_Toc66867169)

[Supplemental Table IV. 10](#_Toc66867170)

[2. Supplemental Figures 13](#_Toc66867171)

[Supplemental Figures I 14](#_Toc66867172)

[Supplemental Figure II 15](#_Toc66867173)

[Supplemental Figure III 16](#_Toc66867174)

[Supplemental Figure IV 17](#_Toc66867175)

[Supplemental Figure V 18](#_Toc66867176)

# 1. Supplemental Tables

Supplemental Table I. **TaqMan probes used in the qRT-PCR.**

| Gene | Probe |
| --- | --- |
| BANP | Hs0099999904_m1 |
| PDCD6IP | Hs00994345_m1 |
| CAMK2G | Hs00968809_mH |
| E2F4 | Hs00608098_m1 |
| PPIA | Hs0099999904_m1 |

Supplemental Table II. **Proportion of negative Z-score regarding the five neuropsychological domains adjusted by age and educational level.** The neuropsychological tests were performed in five CADASIL patients from the validation cohort.

|  | Percentage of negative Z-score |
| --- | --- |
| Executive Function | 80% (4/5) |
| Attention and Information Processing Speed | 60% (3/5) |
| Motor Speed | 60% (3/5) |
| Visuoconstructional Skills | 20% (1/5) |
| Verbal Memory | 60% (3/5) |
| Working Memory | 60% (3/5) |

Supplemental Table III. **List of the GO terms enriched in the category of Biological Process.**

| ID | Description | Set Size | NES | p-value | p.adjust |
| --- | --- | --- | --- | --- | --- |
| GO:0006082 | organic acid metabolic process | 482 | -1.47 | 1.01x10^-3^ | 8.49x10^-2^ |
| [GO:0007155](http://amigo.geneontology.org/amigo/term/GO:0007155) | cell adhesion | 480 | -1.41 | 1.01x10^-3^ | 8.49x10^-2^ |
| GO:0009968 | negative regulation of signal transduction | 461 | -1.39 | 1.01x10^-3^ | 8.49x10^-2^ |
| GO:0010648 | negative regulation of cell communication | 497 | -1.37 | 1.01x10^-3^ | 8.49x10^-2^ |
| GO:0023057 | negative regulation of signaling | 497 | -1.37 | 1.01x10^-3^ | 8.49x10^-2^ |
| GO:1901698 | response to nitrogen compound | 424 | -1.49 | 1.01x10^-3^ | 8.49x10^-2^ |
| GO:0009628 | response to abiotic stimulus | 462 | -1.40 | 1.01x10^-3^ | 8.49x10^-2^ |
| GO:0022610 | biological adhesion | 484 | -1.41 | 1.01x10^-3^ | 8.49x10^-2^ |
| GO:0030182 | neuron differentiation | 472 | -1.37 | 1.01x10^-3^ | 8.49x10^-2^ |
| GO:1901135 | carbohydrate derivative metabolic process | 484 | -1.49 | 1.01x10^-3^ | 8.49x10^-2^ |
| GO:0019752 | carboxylic acid metabolic process | 449 | -1.45 | 1.01x10^-3^ | 8.49x10^-2^ |
| GO:0043436 | oxoacid metabolic process | 476 | -1.49 | 1.01x10^-3^ | 8.49x10^-2^ |
| GO:0010243 | response to organonitrogen compound | 398 | -1.47 | 1.01x10^-3^ | 8.49x10^-2^ |
| GO:0040012 | regulation of locomotion | 393 | -1.47 | 1.01x10^-3^ | 8.49x10^-2^ |
| GO:0051270 | regulation of cellular component movement | 401 | -1.5 | 1.01x10^-3^ | 8.49x10^-2^ |
| GO:0072359 | circulatory system development | 385 | -1.55 | 1.01x10^-3^ | 8.49x10^-2^ |
| GO:0009894 | regulation of catabolic process | 370 | -1.59 | 1.01x10^-3^ | 8.49x10^-2^ |
| GO:0034097 | response to cytokine | 381 | -1.44 | 1.01x10^-3^ | 8.49x10^-2^ |
| GO:0035295 | tube development | 381 | -1.56 | 1.01x10^-3^ | 8.49x10^-2^ |
| GO:0048878 | chemical homeostasis | 409 | -1.43 | 1.01x10^-3^ | 8.49x10^-2^ |
| GO:0060548 | negative regulation of cell death | 380 | -1.43 | 1.01x10^-3^ | 8.49x10^-2^ |
| GO:0030334 | regulation of cell migration | 354 | -1.55 | 1.01x10^-3^ | 8.49x10^-2^ |
| GO:0032787 | monocarboxylic acid metabolic process | 279 | -1.6 | 1.02x10^-3^ | 8.49x10^-2^ |
| GO:1901137 | carbohydrate derivative biosynthetic process | 279 | -1.53 | 1.02x10^-3^ | 8.49x10^-2^ |
| GO:0035239 | tube morphogenesis | 309 | -1.54 | 1.02x10^-3^ | 8.49x10^-2^ |
| GO:0090407 | organophosphate biosynthetic process | 280 | -1.52 | 1.02x10^-3^ | 8.49x10^-2^ |
| GO:0031329 | regulation of cellular catabolic process | 320 | -1.53 | 1.03x10^-3^ | 8.49x10^-2^ |
| GO:0032870 | cellular response to hormone stimulus | 277 | -1.54 | 1.03x10^-3^ | 8.49x10^-2^ |
| GO:0072358 | cardiovascular system development | 264 | -1.55 | 1.03x10^-3^ | 8.49x10^-2^ |
| GO:0071417 | cellular response to organonitrogen compound | 236 | -1.55 | 1.03x10^-3^ | 8.49x10^-2^ |
| GO:1901699 | cellular response to nitrogen compound | 254 | -1.54 | 1.03x10^-3^ | 8.49x10^-2^ |
| GO:0001944 | vasculature development | 260 | -1.56 | 1.03x10^-3^ | 8.49x10^-2^ |
| GO:0001568 | blood vessel development | 252 | -1.54 | 1.03x10^-3^ | 8.49x10^-2^ |
| GO:0048514 | blood vessel morphogenesis | 221 | -1.53 | 1.04x10^-3^ | 8.49x10^-2^ |
| GO:0030335 | positive regulation of cell migration | 207 | -1.6 | 1.05x10^-3^ | 8.49x10^-2^ |
| GO:0006979 | response to oxidative stress | 196 | -1.6 | 1.05x10^-3^ | 8.49x10^-2^ |
| GO:0044057 | regulation of system process | 175 | -1.61 | 1.05x10^-3^ | 8.49x10^-2^ |
| GO:0009896 | positive regulation of catabolic process | 185 | -1.65 | 1.06x10^-3^ | 8.49x10^-2^ |
| GO:0048193 | Golgi vesicle transport | 169 | -1.65 | 1.06x10^-3^ | 8.49x10^-2^ |
| GO:0001667 | ameboidal-type cell migration | 162 | -1.64 | 1.06x10^-3^ | 8.49x10^-2^ |
| GO:0035690 | cellular response to drug | 145 | -1.67 | 1.07x10^-3^ | 8.49x10^-2^ |
| GO:0031331 | positive regulation of cellular catabolic process | 158 | -1.56 | 1.07x10^-3^ | 8.49x10^-2^ |
| GO:0071496 | cellular response to external stimulus | 158 | -1.57 | 1.07x10^-3^ | 8.49x10^-2^ |
| GO:1901293 | nucleoside phosphate biosynthetic process | 144 | -1.61 | 1.07x10^-3^ | 8.49x10^-2^ |
| GO:0036293 | response to decreased oxygen levels | 140 | -1.57 | 1.07x10^-3^ | 8.49x10^-2^ |
| GO:0040013 | negative regulation of locomotion | 139 | -1.57 | 1.07x10^-3^ | 8.49x10^-2^ |
| GO:0050900 | leukocyte migration | 140 | -1.56 | 1.07x10^-3^ | 8.49x10^-2^ |
| GO:0051271 | negative regulation of cellular component movement | 139 | -1.57 | 1.07x10^-3^ | 8.49x10^-2^ |
| GO:0001101 | response to acid chemical | 148 | -1.66 | 1.07x10^-3^ | 8.49x10^-2^ |
| GO:0070482 | response to oxygen levels | 146 | -1.59 | 1.07x10^-3^ | 8.49x10^-2^ |
| GO:0010038 | response to metal ion | 149 | -1.57 | 1.07x10^-3^ | 8.49x10^-2^ |
| GO:0009165 | nucleotide biosynthetic process | 143 | -1.61 | 1.07x10^-3^ | 8.49x10^-2^ |
| GO:0070997 | neuron death | 137 | -1.7 | 1.07x10^-3^ | 8.49x10^-2^ |
| GO:0016236 | macroautophagy | 136 | -1.52 | 1.08x10^-3^ | 8.49x10^-2^ |
| GO:0001655 | urogenital system development | 133 | -1.55 | 1.08x10^-3^ | 8.49x10^-2^ |
| GO:0001666 | response to hypoxia | 133 | -1.57 | 1.08x10^-3^ | 8.49x10^-2^ |
| GO:0010506 | regulation of autophagy | 135 | -1.67 | 1.08x10^-3^ | 8.49x10^-2^ |
| GO:0072330 | monocarboxylic acid biosynthetic process | 131 | -1.53 | 1.08x10^-3^ | 8.49x10^-2^ |
| GO:0031668 | cellular response to extracellular stimulus | 129 | -1.56 | 1.08x10^-3^ | 8.49x10^-2^ |
| GO:0034976 | response to endoplasmic reticulum stress | 128 | -1.63 | 1.08x10^-3^ | 8.49x10^-2^ |

NES: normalized enrichment score; p.adjust: adjusted p-value.

Supplemental Table IV. **List of the GO terms enriched in the category of Cellular Components.**

| ID | Description | Set Size | NES | p-value | p.adjust |
| --- | --- | --- | --- | --- | --- |
| GO:0005789 | endoplasmic reticulum membrane | 472 | -1.38 | 1.00x10^-3^ | 5.03x10^-2^ |
| GO:0030054 | cell junction | 472 | -1.58 | 1.00x10^-3^ | 5.03x10^-2^ |
| GO:0031226 | intrinsic component of plasma membrane | 470 | -1.42 | 1.00x10^-3^ | 5.03x10^-2^ |
| GO:0005768 | endosome | 368 | -1.45 | 1.01x10^-3^ | 5.03x10^-2^ |
| GO:0005773 | vacuole | 338 | -1.48 | 1.01x10^-3^ | 5.03x10^-2^ |
| GO:0005887 | integral component of plasma membrane | 445 | -1.47 | 1.01x10^-3^ | 5.03x10^-2^ |
| GO:0098827 | endoplasmic reticulum subcompartment | 475 | -1.38 | 1.01x10^-3^ | 5.03x10^-2^ |
| GO:0044431 | Golgi apparatus part | 403 | -1.5 | 1.01x10^-3^ | 5.03x10^-2^ |
| GO:0098791 | Golgi subcompartment | 365 | -1.55 | 1.01x10^-3^ | 5.03x10^-2^ |
| GO:0098590 | plasma membrane region | 396 | -1.54 | 1.01x10^-3^ | 5.03x10^-2^ |
| GO:0098796 | membrane protein complex | 428 | -1.41 | 1.01x10^-3^ | 5.03x10^-2^ |
| GO:0012506 | vesicle membrane | 318 | -1.6 | 1.01x10^-3^ | 5.03x10^-2^ |
| GO:0030659 | cytoplasmic vesicle membrane | 307 | -1.61 | 1.01x10^-3^ | 5.03x10^-2^ |
| GO:0000139 | Golgi membrane | 310 | -1.49 | 1.01x10^-3^ | 5.03x10^-2^ |
| GO:0044437 | vacuolar part | 257 | -1.59 | 1.02x10^-3^ | 5.03x10^-2^ |
| GO:0070161 | anchoring junction | 227 | -1.62 | 1.04x10^-3^ | 5.03x10^-2^ |
| GO:0005912 | adherens junction | 220 | -1.66 | 1.04x10^-3^ | 5.03x10^-2^ |
| GO:0005911 | cell-cell junction | 173 | -1.7 | 1.06x10^-3^ | 5.03x10^-2^ |
| GO:0030055 | cell-substrate junction | 175 | -1.63 | 1.06x10^-3^ | 5.03x10^-2^ |
| GO:0005924 | cell-substrate adherens junction | 172 | -1.67 | 1.06x10^-3^ | 5.03x10^-2^ |
| GO:0005925 | focal adhesion | 172 | -1.67 | 1.06x10^-3^ | 5.03x10^-2^ |
| GO:0030139 | endocytic vesicle | 128 | -1.64 | 1.08x10^-3^ | 5.03x10^-2^ |
| GO:003066 | endocytic vesicle membrane | 80 | -1.73 | 1.15x10^-3^ | 5.14x10^-2^ |
| GO:0001739 | sex chromatin | 3 | -1.73 | 1.77x10^-3^ | 7.54x10^-2^ |
| GO:0042175 | nuclear outer membrane-endoplasmic reticulum membrane network | 482 | -1.38 | 2.01x10^-3^ | 7.69x10^-2^ |
| GO:0000323 | lytic vacuole | 297 | -1.45 | 2.03x10^-3^ | 7.69x10^-2^ |
| GO:0005764 | lysosome | 297 | -1.45 | 2.03x10^-3^ | 7.69x10^-2^ |
| GO:0005774 | vacuolar membrane | 181 | -1.54 | 2.12x10^-3^ | 7.75x10^-2^ |
| GO:0005802 | trans-Golgi network | 108 | -1.6 | 2.20x10^-3^ | 7.76x10^-2^ |
| GO:0030134 | COPII-coated ER to Golgi transport vesicle | 48 | -1.82 | 2.45x10^-3^ | 8.36x10^-2^ |
| GO:0033176 | proton-transporting V-type ATPase complex | 10 | -1.79 | 2.93x10^-3^ | 9.67x10^-2^ |
| GO:0030133 | transport vesicle | 155 | -1.52 | 3.21x10^-3^ | 1.03x10^-1^ |
| GO:0044853 | plasma membrane raft | 40 | -1.77 | 3.71x10^-3^ | 1.15x10^-1^ |
| GO:0005901 | caveola | 32 | -1.87 | 3.92x10^-3^ | 1.15x10^-1^ |
| GO:0016328 | lateral plasma membrane | 31 | -1.78 | 3.92x10^-3^ | 1.15x10^-1^ |
| GO:0001533 | cornified envelope | 35 | 1.96 | 4.29x10^-3^ | 1.19x10^-1^ |
| GO:0062023 | collagen-containing extracellular matrix | 131 | -1.57 | 4.30x10^-3^ | 1.19x10^-1^ |
| GO:0005798 | Golgi-associated vesicle | 80 | -1.59 | 4.62x10^-3^ | 1.24x10^-1^ |
| GO:0031012 | extracellular matrix | 173 | -1.51 | 5.29x10^-3^ | 1.24x10^-1^ |
| GO:0043235 | receptor complex | 134 | -1.52 | 5.39x10^-3^ | 1.33x10^-1^ |
| GO:0016324 | apical plasma membrane | 115 | -1.55 | 5.49x10^-3^ | 1.33x10^-1^ |
| GO:0030061 | mitochondrial crista | 6 | 1.78 | 5.71x10^-3^ | 1.33x10^-1^ |
| GO:0031314 | extrinsic component of mitochondrial inner membrane | 6 | 1.8 | 5.71x10^-3^ | 1.33x10^-1^ |
| GO:0031414 | N-terminal protein acetyltransferase complex | 6 | 1.78 | 5.71x10^-3^ | 1.33x10^-1^ |
| GO:0016471 | vacuolar proton-transporting V-type ATPase complex | 7 | -1.81 | 5.90x10^-3^ | 1.34x10^-1^ |
| GO:0031092 | platelet alpha granule membrane | 10 | 1.79 | 6.27x10^-3^ | 1.36x10^-1^ |
| GO:0031252 | cell leading edge | 168 | -1.47 | 6.36x10^-3^ | 1.36x10^-1^ |
| GO:0097060 | synaptic membrane | 122 | -1.55 | 6.49x10^-3^ | 1.36x10^-1^ |
| GO:0009986 | cell surface | 261 | -1.4 | 7.16x10^-3^ | 1.36x10^-1^ |
| GO:0044440 | endosomal part | 229 | -1.4 | 7.24x10^-3^ | 1.36x10^-1^ |
| GO:0098797 | plasma membrane protein complex | 185 | -1.48 | 7.38x10^-3^ | 1.36x10^-1^ |
| GO:0098794 | postsynapse | 188 | -1.47 | 7.40x10^-3^ | 1.36x10^-1^ |
| GO:0044291 | cell-cell contact zone | 32 | -1.64 | 7.83x10^-3^ | 1.36x10^-1^ |
| GO:0016323 | basolateral plasma membrane | 95 | -1.52 | 7.88x10^-3^ | 1.36x10^-1^ |
| GO:0031093 | platelet alpha granule lumen | 26 | -1.71 | 7.96x10^-3^ | 1.36x10^-1^ |
| GO:0014704 | intercalated disc | 23 | -1.74 | 7.16x10^-3^ | 1.36x10^-1^ |
| GO:0005766 | primary lysosome | 69 | -1.57 | 7.16x10^-3^ | 1.36x10^-1^ |
| GO:0042582 | azurophil granule | 69 | -1.57 | 7.16x10^-3^ | 1.36x10^-1^ |
| GO:0098552 | side of membrane | 178 | -1.44 | 7.16x10^-3^ | 1.36x10^-1^ |
| GO:0060076 | excitatory synapse | 15 | -1.72 | 7.16x10^-3^ | 1.36x10^-1^ |

NES: normalized enrichment score; p.adjust: adjusted p-value.

# 2. Supplemental Figures

**Supplemental Figures I:** Z-score regarding the five neuropsychological domains adjusted by age and educational level, evaluated in the five patients with available cognitive information.

EF: executive function; IPS: information processing speed; VCF: visuoconstructional function.

**Supplemental Figure II.** *E2F4* gene expression profile in decreasing order (GTEx Portal, https://gtexportal.org/home/gene/E2F4.

**Supplemental Figure III.** Single-Nuclei Brain RNA-seq expression of *E2F4*.

**Supplemental Figure IV.** Enrichment map (biological process) of the top 60 terms. This map groups gene ontology (GO) terms by similarity. Nodes are colored by p-value and their size reflects the number of genes found in that term.

**Supplemental Figure V.** Enrichment map (cellular components) of the top 60 terms. This map groups gene ontology (GO) terms by similarity. Nodes are colored by p-value and their size reflects the number of genes found in that term.

## Supplemental Figures I


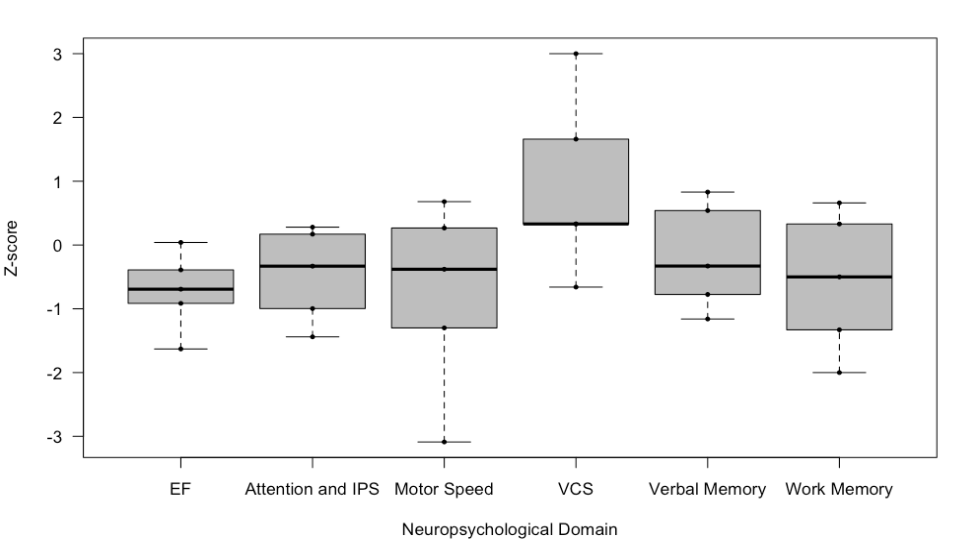


## Supplemental Figure II


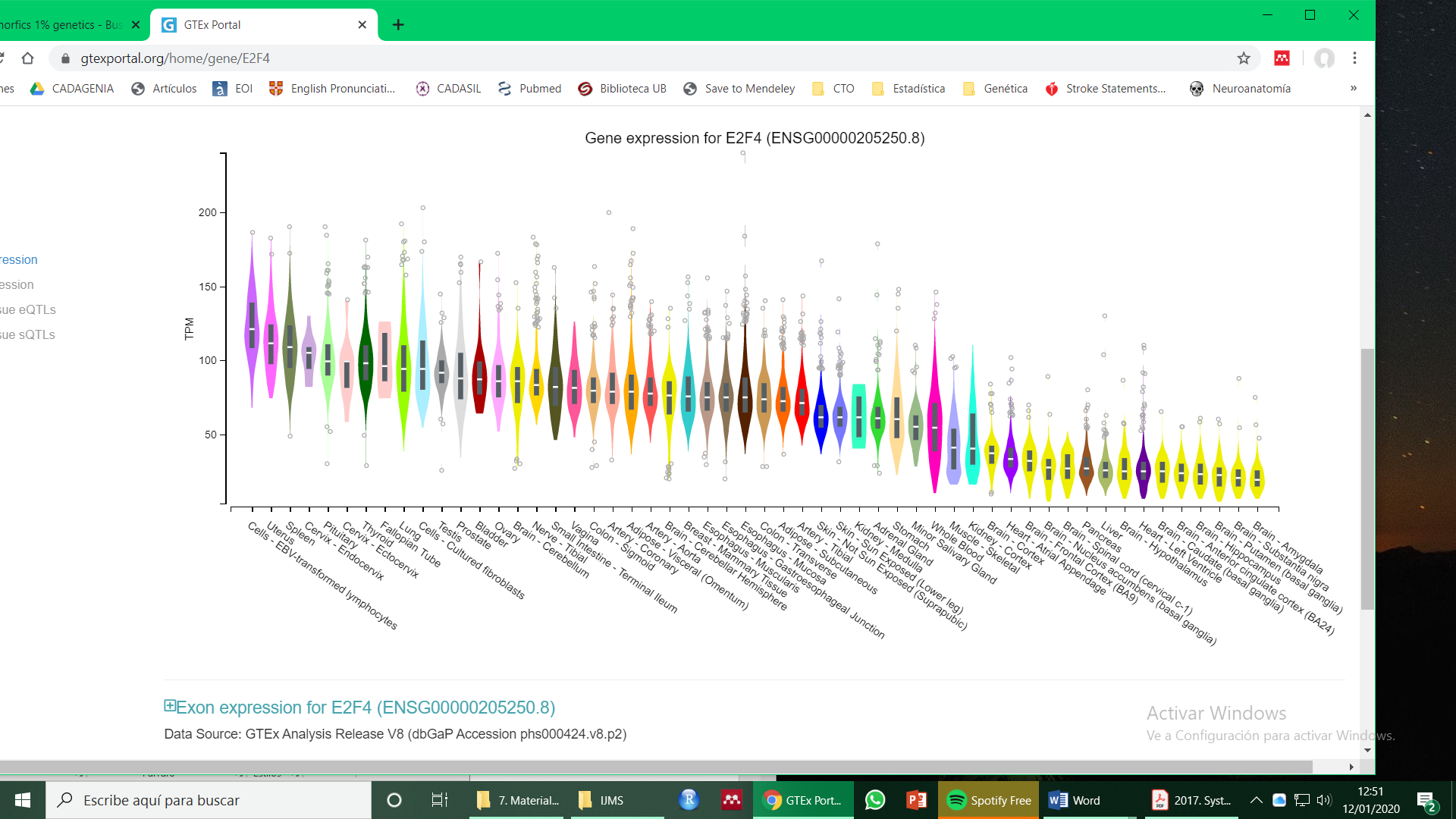


## Supplemental Figure III


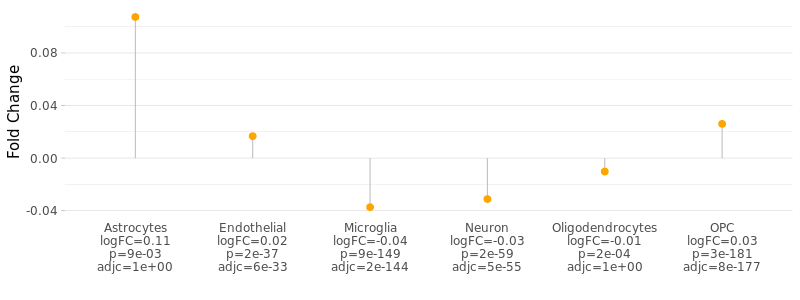


## Supplemental Figure IV

## Supplemental Figure V
